# Reproductive Safety Issues of Novel Small Molecules for the Treatment of Inflammatory Bowel Disease: A Systematic Review

**DOI:** 10.3390/jcm13010034

**Published:** 2023-12-20

**Authors:** Niloufar Monfared, Matthew Gold, Isabel Carbery, Robyn Laube, Christian P. Selinger

**Affiliations:** Leeds Gastroenterology Institute, Leeds Teaching Hospitals, Leeds LS9 7TF, UK; niloufar.monfared@nhs.net (N.M.); matthew.gold1@nhs.net (M.G.); robynlaube@hotmail.com (R.L.)

**Keywords:** inflammatory bowel disease, novel small molecule, pregnancy

## Abstract

Maintenance of remission during pregnancy is vital for women with inflammatory bowel disease (IBD). The antenatal safety of novel small molecules for IBD is yet to be ascertained. We aimed to describe the current evidence on reproductive data regarding small-molecule drugs. We performed a systematic review searching Embase Classic + Embase and Ovid MEDLINE for reproductive outcomes for tofacitinib, filgotinib, upadacitininb, and ozanimod. Additionally, we asked the manufacturers for available data on file regarding reproduction. We analysed data from 10 sources; six studies and four manufacturer reports were identified from our search. Significant malformation risks were reported for tofacitinib, filgotinib, upadacitininb, and ozanimod in animal studies. In 126 tofacitinib-exposed pregnancies, there were 55 live births with 2 congenital malformations and 1 serious infant infection, 14 terminations, 15 miscarriages, and 42 outcomes unknown. In 50 filgotinib-exposed pregnancies, there were 20 healthy babies, 1 congenital malformation, 9 terminations, 10 miscarriages, and 10 outcomes unknown. In 78 upadacitinib-exposed pregnancies, there were 30 healthy babies, 15 terminations, 15 miscarriages, and 18 outcomes unknown. In 60 ozanimod-exposed pregnancies, there were 31 live births with 1 congenital malformation, 1 case of intra-uterine growth restriction, 1 case of neonatal icterus, 13 terminations, 9 miscarriages, and 8 unknown outcomes. Animal data suggest significant risks of malformations for tofacitinib, filgotinib, upadacitininb, and ozanimod. Human data from clinical trials and real-world observations do not show concerning data so far, but these are very limited. Currently, alternative treatments should be used for IBD during pregnancy.

## 1. Introduction

Crohn’s disease (CD) and ulcerative colitis (UC) are the two main forms of inflammatory bowel disease (IBD) [1]. The global prevalence of IBD has risen from 79.5 per 100,000 in 1990 to 84.3 per 100,000 in 2017 [2]. This increase has been seen largely in newly industrialising economies, such as Africa and Asia, with rates stabilising or decreasing in Europe and the USA [3]. The peak incidence of IBD occurs between 20 and 30 years of age, which coincides with peak fertility and family planning [4,5]. In addition to maternal symptoms, active IBD is associated with a significant risk of adverse pregnancy outcomes, including increased rates of preterm birth, intrauterine growth retardation, and spontaneous abortion [6,7]. The most recent European Crohn’s and Colitis Organisation (ECCO) guidelines highlight the need to establish remission prior to conception and to maintain remission throughout pregnancy [8], and understanding how to do this safely is paramount to providing good antenatal IBD care.

The antenatal safety profile for traditional oral medical treatments used for IBD (corticosteroids, mesalazine, methotrexate, and thiopurines) is well established [8,9,10,11]. Methotrexate is contraindicated during pregnancy [8]. The use of corticosteroids (when clinically required for flare management), mesalazine, and thiopurines is encouraged during pregnancy as the benefits far outweigh the small potential risks [8]. Biologics, including infliximab, adalimumab, golimumab, vedolizumab, and ustekinumab, are large immunoglobulins that do not cross the placenta passively but are actively transported during the second and third trimesters. Therefore, infants exposed to these molecules in utero are born with significant biologic exposure; however, there is no significant exposure of the foetus during the organ-forming phase of the first trimester. Large-scale observation studies have shown reassuring data on the maternal and foetal safety of these biologics used in the treatment of IBD [8,12,13].

Small-molecule drugs (SMDs) include Janus kinase (JAK) inhibitors, sphingosine-1-phosphate (S1P) receptor modulators, and phosphodiesterase four inhibitors. These all act on specific molecular pathways to modulate immunological function [14]. The current SMDs used to treat IBD include tofacitinib, filgotinib, upadacitinib, and ozanimod [14].

SMDs, as their name suggests, are of a low molecular weight and lack complex structure [15]. The benefit of this is that SMDs resist gastric degradation and can be administered orally [16]. However, this also means that in contrast to biological agents, small molecules cross the placenta passively during all phases of pregnancy; therefore, it is vital to establish the reproductive safety profile of these small-molecule drugs.

We aim to perform a systematic review to summarise the current safety profile surrounding maternal exposure to small molecules to inform both patients and clinicians in choosing the correct drug treatment for pregnant patients with IBD. 

## 2. Materials and Methods

This systematic review follows the preferred reporting items for systematic reviews and meta-analyses (PRISMA) recommendations (see Supplementray Files) [17]. The protocol was prospectively registered with PROSPERO (reference CRD42023469582). Human pregnancy outcomes of interest included congenital malformations, immediate post-partum infant issues, complications of pregnancy, terminations, miscarriages, and stillbirths. In addition, we aimed to describe pre-clinical reproductive animal data as ascertained directly from pharmaceutical manufacturers (Pfizer [18], AbbVie [19], Galapagos Global [20], and Bristol-Myers Squibb [21]).

### 2.1. Eligibility Criteria

Our inclusion criteria for studies identified in the systematic review required any pregnancy exposure to a SMD of interest (tofacitinib, ozanimod, filgotinib, upadacitinib), pregnancy outcomes to be reported (live births, congenital malformations, miscarriages, medical terminations, other complications—for example immediate post-natal issues), and the full text articles to be available in English. Publications were excluded if they were not in English, or if no safety outcomes were reported. 

### 2.2. Literature Search

The authors (IC, CPS, NM, MG) developed a comprehensive search strategy which was inputted into electronic databases including Embase Classic + Embase (1947 to 10th 2023) and Ovid MEDLINE(R) ALL (1946 to 10 May 2023). The search was conducted on the 11 May 2023 using a full text search. The search terms used for IBD included “Crohn*”, “Ulcerative Coliti*”, UC, “Coliti*”, Ileitis, IBD, Inflammatory bowel, exp inflammatory bowel diseases. For pregnancy, terms used were “breast fe$”, “breast-fe$”, “breast milk”, “pregnan$”, “lactation”, “infant”, “birth”, “congenital”, “new born”, “foetus”, “foetal”, “fetus” “fetal”. We also approached each pharmaceutical manufacturer (Pfizer, AbbVie, Galapagos and Bristol-Myers-Squibb) regarding any preclinical and human reproductive safety data available from preclinical and clinical developments programs as well as post-marketing pharmacovigilance by approaching their medical information departments via email. The effect measure of interest was the proportion of outcomes relative to reported pregnancies.

### 2.3. Selection of Studies

Two members of the team (NM and MJG) independently reviewed and assessed the abstracts and titles from the initial search. If one reviewer deemed a study eligible, the full text was assessed by both reviewers, and a consensus was reached along with input from CS and IC. Where possible, only pregnancy outcomes associated with maternal exposure to SMD monotherapy were used.

### 2.4. Data Extraction and Synthesis

Data extraction was performed by NM and MG. The following data was extracted: number of pregnancies, number of live births, healthy births, congenital malformations, miscarriages, medical terminations, other complications, and loss to follow-up. Data were entered into tables, and reports were checked for overlaps between different sources. Any overlapping clinical trial data were excluded at this stage, ensuring no duplication of reported data. Pre-clinical data were described as reported by the manufacturers.

### 2.5. Risk of Bias Assessment

As the results were derived from non-randomised studies, two reviewers (NM and MJG) independently assessed the ten studies available using the Newcastle–Ottawa scale (NOS). The NOS is a widely used scoring system to assess the risk of bias in non-randomised studies. The scores are derived from three domains (selection, comparability, and exposure), ranging between 0 and 9, with higher scores having the least risk of bias.

## 3. Results

**Figure 1:**
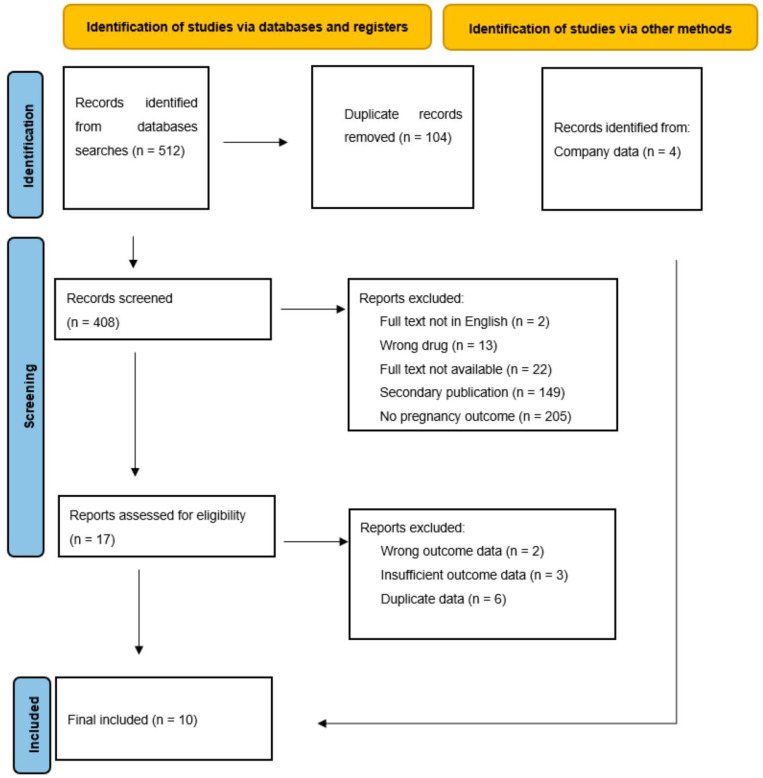
PRISMA diagram [17]. Systematic search and study selection process.

The search terms yielded 512 results, from which six publications were identified and used for data extraction. Data were also received from all four manufacturers (Figure 1). Overall, from the 10 data sources (Table 1), 314 pregnancies (315 pregnancy outcomes due to one twin pregnancy) were included, resulting in 130 healthy live births (41%), 51 medical terminations (16%), 49 miscarriages (16%), 4 congenital malformations, and 3 other complications (2%), with 78 lost to follow-up (25%; Table 2). 

Table 1 details the number of pregnancies per drug; sources include a case report, cohort studies, clinical trial reports, conference abstracts, and drug safety information requested and provided directly from pharmaceutical companies. Pregnancy outcomes are summarised for each drug in Table 2.

### 3.1. Tofacitinib

Pre-clinical animal data (rats and rabbits) revealed that tofacitinib is teratogenic and negatively affects female fertility (decreased pregnancy rate, which decreases in the numbers of corpora lutea, implantation sites, and viable foetuses, and an increase in early miscarriages). Tofacitinib had no effects on male fertility, sperm motility, or sperm concentration.

There were 126 pregnancies in total for tofacitinib, 14 medical terminations, 15 miscarriages, and 55 live births, with 52 of these being normal, healthy births. Congenital malformations for tofacitinib included one ventricular septal defect [27] and one pulmonary valve stenosis [18,23]. Other complications included one incidence of a “serious infection”, which occurred in the first year of life [22]. A total of 42 pregnancy outcomes were lost to follow-up or unknown. Of all reported pregnancies, 19 were combined with methotrexate/tofacitinib therapy [18], and the pregnancy outcomes quoted from the Pfizer database did not differentiate between monotherapy and combination therapy. 

### 3.2. Filgotinib

Embryo–foetal development studies in rats and rabbits demonstrated embryolethality and teratogenicity at exposures comparable to 200 mg filgotinib dosing in humans. Visceral and skeletal malformations and/or variations were observed at all dose levels of filgotinib, including in rats (internal hydrocephaly, dilated ureters, and multiple vertebral anomalies) and rabbits (visceral malformations mainly in the lungs and cardiovascular system and skeletal malformations).

A total of 50 pregnancies were included for filgotinib. Pregnancy outcomes included 9 medical terminations, 10 miscarriages, and 21 live births, of which 20 were normal healthy babies. One congenital malformation was a case of Pentalogy of Fallot, which required corrective surgery at two months post-partum [20]. There were no other complications reported. Ten pregnancies were lost to follow-up, or their outcomes are unknown. 

### 3.3. Upadacitinib

Pre-clinical animal studies revealed upadacitinib to be teratogenic in rats and rabbits. Increases in skeletal malformations in rats at 1.6, 0.8, and 0.6 times the clinical exposure at the 15, 30, and 45 mg doses were found. In rabbits, increases in cardiovascular malformations were observed at 15, 7.6, and 5.6 times the clinical exposure at the 15, 30, and 45 mg doses, respectively. 

A total of 78 pregnancies were reported for upadacitinib: 15 medical terminations, 15 miscarriages, and 30 live births, of which all 30 were healthy babies. Two premature births were recorded among these 30 healthy live births, as they were otherwise known to have progressed well [19]. There were no reported congenital malformations or other complications, and 18 of the pregnancies were lost to follow-up. Ten of the 78 pregnancies were exposed to a combination of upadacitinib and methotrexate. AbbVie specified that the ten pregnancies exposed to upadacitinib/methotrexate combination therapy all had the pregnancy outcome of miscarriage [19].

### 3.4. Ozanimod

Embryo–foetal development was adversely affected with low (rats) or no (rabbits) safety margins resulting in embryolethality and teratogenicity (generalised oedema/anasarca and malpositioned testes in rats; malpositioned caudal vertebrae and malformations of the great vessels in rabbits).

For ozanimod, there were 61 pregnancy outcomes for 60 total pregnancies due to one twin pregnancy. There were 13 medical terminations, 9 miscarriages, and 31 live births; 28 were healthy live births, including three premature births that progressed without complication [27]. One congenital malformation of a duplex kidney was reported for ozanimod [27], and other complications included one incidence of intra-uterine growth restriction resolving in the first year of life [27,28] and one incidence of neonatal icterus [27]. Eight pregnancy outcomes were lost to follow-up or remain unknown.

### 3.5. Risk of Bias Assessment

Using the NOS, 10 studies were evaluated for bias. The two cohort studies were deemed to be at moderate risk of bias. The remaining eight studies were high-risk (Table 3; four data sources were directly from pharmaceutical companies, one was a case report, one was a conference abstract, and the remaining two used clinical trial data). 

## 4. Discussion

With the increasing use of SMDs for IBD [29], there is an urgent need to understand their reproductive safety profile to inform clinicians and patients regarding their use in women of reproductive age [30]. In this systematic review, we summarised animal data for tofacitinib, filgotinib, upadacitnib, and ozanimod. Significant risks of loss of pregnancy and severe congenital malformations were found for each drug at doses comparable to clinical use in humans. The available human data suggest that 41% of the total pregnancies exposed to SMDs resulted in healthy live births. A number of malformations were reported, but this is probably in line with expectations, as congenital malformations occur in 3–7% of pregnancies in the general population [31].

To understand how much reassurance can be drawn from the available data, we must critically reflect on the outcomes reported and the actual drug exposure. First, there was a high number of medical terminations and pregnancies with an unknown outcome, which increases bias towards better pregnancy outcomes in the reports. Second, for any pregnancies arising from the clinical trials program the likely exposure to the medication will be very short due to a number of reasons specific to clinical trials. Patients participating in clinical trials of medications in development are tested for pregnancy very regularly, and if a pregnancy is found, medication exposure ceases immediately. Furthermore, patients exposed during the immediate pre-conception phase but possibly not during early pregnancy are often also included in the reported data. Therefore, we cannot be sure of the extent of first-trimester exposure in the reported pregnancies. These limitations must be considered when assessing the available human exposure data.

Where possible, only pregnancy outcomes exposed to SMD monotherapy were used. The one exception is data from Pfizer, which had 19 pregnancies exposed to both methotrexate and tofacitinib without specifications on which pregnancy outcomes were due tofacitinib monotherapy or the combined methotrexate–tofacitinib therapy [18]. AbbVie highlighted that 10 out of 78 total pregnancies were also exposed to a combination of methotrexate and upadacitinib but clarified that all 10 of these combined exposure cases’ outcome was miscarriage, with the rest of the 68 pregnancy outcomes being associated with upadacitinib monotherapy exposure only [19].

The event rates of miscarriage, malformations, and other pregnancy outcomes are not unexpected for patients with IBD. For example, biologic agents were associated with 13% of miscarriages in a meta-analysis of 11,172 pregnancies [32], compared to the data reported here, with a 16% incidence of miscarriage in 314 pregnancies. Similarly, in the meta-analysis of biologics in pregnancy by Nielsen et al., 11% of exposed pregnancies led to miscarriage when associated with biologic use, compared to a 14% prevalence of miscarriage in the general population [33]. This is not a large variation from our review. 

Data on congenital malformations reported in our data set are at an expected level, usually seen in IBD and in the general population. O’Byrne et al. found that for TNF-α inhibitors, there was still no definitive statistical link between exposure to biologics and the development of a congenital abnormality and that its incidence was 4% overall [32]. Similarly, a meta-analysis of pregnancies exposed to biologics used in psoriasis indicated that 3% reported congenital abnormalities [34]. 

Overall, this review highlights the limited literature available on SMD exposure in pregnancy. There is no definitive evidence for SMDs yielding better or worse pregnancy outcomes compared to biologics or, indeed, the general population in terms of miscarriage, congenital malformations, or serious complications [35]. We must emphasise the difference in exposure length likely seen in clinical trial programs (where most SMD data arise from) and during real-world treatment of pregnant IBD patients (where most biologics data stem from). Hence, no firm conclusions can be drawn from the presented limited data on malformations.

There were insufficient data reported on infant infection in this dataset to draw meaningful conclusions. This review found one reported incidence of serious neonatal infection in tofacitinib (out of 74 tofacitinib-exposed pregnancies with a live birth), compared to 2–5% of biologic-exposed IBD pregnancies [32].

As 25% of the pregnancies from this review were lost to follow-up, it should be noted that the number of pregnancy outcomes in the other categories may be under-represented due to the nature of participants and the predisposition for small-molecule exposure to be accidental. It is only clear from this review that 41% of the pregnancy outcomes were healthy live births, leaving a significant level of outcome uncertainty when considering the potential risk of SMDs in pregnancy. However, for the foreseeable future, any human data collated will continue to be related to accidental exposure, so further data may evolve slower than previously seen with IBD biologics.

Given the concerns over teratogenicity with SMDs, clinicians and patients must rely on other IBD therapies with an established and acceptable safety profile for conception, pregnancy, and lactation [8]. Maintenance of remission prior to and during conception is vital to reduce intrapartum flares and the associated risk of adverse pregnancy outcomes [8,13]. Mesalazine and thiopurines have a long-established safety profile and are recommended to be continued during pregnancy and lactation [8]. Anti-TNF biologics have been shown to effectively control disease without any increase in adverse pregnancy outcomes [12,13]. Emerging data for vedolizumab [36] and ustekinumab [13] are so far reassuring for pregnancy. Clinicians should rely on those agents in women contemplating pregnancy.

The strength of our work lies in the rigorous systematic literature search and critical assessment conducted independently by at least two authors with additional data obtained from the manufacturers. Limitations to this review include a relatively small sample size of exposed pregnancies to small molecules. It is also unclear how long the exposure occurred in all the reported cases, at which precise stage of pregnancy, and which outcomes these related to. We did not have access to important maternal health factors such as maternal age, socio-economic status, IBD status, and general health that could confound pregnancy outcomes. As some of the reported pregnancies were also exposed to methotrexate, we cannot be sure whether this influenced outcomes. Additionally, not all patients had a diagnosis of IBD, but this may not matter as it is unlikely for the monitored outcomes to differ significantly between the other indications. Risk of bias analysis suggested a fairly high risk of bias overall, which was to be expected given the nature of the available datasets. Arguably, the Newcastle–Ottawa scale for risk of bias assessment was not designed for the study types included in our systematic review. Due to the small sample size, loss to follow-up had a disproportionate effect on the data.

## 5. Conclusions

Animal data show concerning teratogenic effects. Human exposure shows results that can be expected in IBD cohorts, but the importance of the findings is limited by minimal drug exposure. It is therefore recommended to avoid SMDs in pregnant IBD patients in favour of medical therapies with an established and acceptable pregnancy risk profile.

## Figures and Tables

**Table 1:** Pregnancy outcomes for each study, total pregnancies per drug, and associated conditions.

Study (*n* = 10)	Data Source	Pregnancies (*n* = 314)	Drug
Pfizer, 2023 Ⴟ [18]	Pharmaceutical company data on file: Clinical Trial Programmes and spontaneous reports	122	Tofacitninib
Vinet, 2019 [22]	Cohort study, Marketscan data	4	Tofacitninib
Clowse, 2016 [23]	Cohort study from tofacitinib randomised control trials All data included in Pfizer, 2023	49	Tofacitninib
Fernandez-Sanchez, 2021 [24]	Case report All data included in Pfizer, 2023	1	Tofacitninib
Mahadevan, 2018 [25]	Interventional study data and spontaneous adverse event reports All data included in Pfizer, 2023	116	Tofacitninib
Mahadevan, 2020 [26]	Conference abstract: Updated Phase 3 OCTAVE clinical trial data All data included in Pfizer, 2023	15	Tofacitninib
Bristol-Myers Squibb, 2023 [21]	Pharmaceutical company: data in file, excludes duplicate data from ozanimod/RMS clinical development programme	60	Ozanimod
Minton 2021 [27]	Ozanimod/RMS clinical development programme data All data included in Bristol–Myers–Squibb, 2023	18	Ozanimod
Galapagos Global 2023 [20]	Pharmaceutical company safety database from clinical trial data and data on file	50	Filgotinib
AbbVie Info 2023 Ⴟ [19]	Pharmaceutical company: data on file and clinical trial reports	78	Upadacitinib

**Table 2:** Pregnancy outcomes following exposure to tofacitinib, ozanimod, filgotinib, and updacitinib.

	Tofacitinib Ⴟ	Ozanimod	Filgotinib	Upadacitinib
**Number of publications included**	6	2	1	1
**Number of pregnancies**	126	60 *	50	78 Ⴟ
**Medical terminations**	14	13	9	15
**Miscarriages**	15	9	10	15 Ⴟ
**Live births** - **Congenital malformations** - **Healthy babies** - **Other complications**	55 2 ‡ 52 1 ¥	31 - 1 ‡ - 28 ∞ - 2 ¥	21 - 1 ‡ - 20 - 0	30 - 0 - 30 ∞ - 0
**Unknown/lost to follow-up**	42	8	10	18

Ⴟ Combined methotrexate exposure. Pfizer includes 19 pregnancies exposed to tofacitinib/methotrexate combination therapy (pregnancy outcomes unclear) [18]. AbbVie includes 10 pregnancies exposed to upadacitinib/methotrexate combination therapy but clarifies all 10 combination pregnancies miscarried [19]. ∞ “Healthy babies” include premature babies who go on to be healthy. This includes three premature births for ozanimod [27] and two premature births for for upadacitinib [19]. ‡ Congenital malformations include, for tofacitinib, one ventricular septal defect [27] and one pulmonary valve stenosis [18,23]. Ozanimod had 1 duplex kidney, which was reported [27]. Filgotinib had 1 case of Pentalogy of Fallot, which required corrective surgery 2 months post-partum [20]. ¥ “Other complications” for tofacitinib include serious infection within the first year of life [22]; for ozanimod, complications include one intra-uterine growth restriction (IUGR) resolving in the first year of life [27,28] and one neonatal icterus [27]. * For ozanimod, there were 61 pregnancy outcomes compared to 60 total pregnancies due to one twin pregnancy.

**Table 3:** Risk of bias assessment.

	Selection				Comparability	Outcome			Totals
Study	Representativeness of Exposed Cohort	Selection of Non-Exposed Cohort	Ascertainment of Exposure	Outcome of Interest Was Not Present at the Start of Study	Comparability of Cohorts on the Basis of Design or Analysis	Assessment of Outcome	Follow up Sufficient for Outcomes to Occur	Adequacy of Follow-Up Cohorts	
AbbVie, 2023 [19]	0	0	0	0	0	0	0	0	0
Bristol-Myers Squibb, 2023 [21]	0	0	0	0	0	0	0	0	0
Clowse et al., 2016 [23]	0	0	1	1	0	1	0	0	3
Fernandez-sanchez et al., 2021 [24]	0	0	0	1	0	1	0	0	2
Galapagos Global, 2023 [20]	0	0	0	0	0	0	0	0	0
Mahadevan et al., 2018 [25]	0	0	1	1	0	0	0	0	2
Mahadevan et al., 2020 [26]	0	0	0	0	0	0	0	0	0
Minton et al., 2021 [27]	0	0	0	0	0	0	0	0	0
Pfizer, 2023 [18]	0	0	0	0	0	0	0	0	0
Vinet et al., 2019 [22]	0	0	1	1	0	1	0	0	3

Risk of bias assessment: The Newcastle–Ottawa scale (NOS) was used for quality assessment of non-randomised studies. A maximum of one star was awarded for each selection and outcome, with a maximum of two stars for comparability. Total scores in case–control studies equate to “Good”: ≥5; “Moderate”: 3–4; “Poor”: ≤2 and for cohort studies, “Good”: ≥7; “Moderate”: 5–6; “Poor”: ≤4.

## Data Availability

Data are contained within the article.

## Supplementary Materials

The following supporting information can be downloaded at: https://www.mdpi.com/article/10.3390/jcm13010034/s1.
